# Four-Point Measurement Setup for Correlative Microscopy of Nanowires

**DOI:** 10.3390/nano13172451

**Published:** 2023-08-30

**Authors:** Bartosz C. Pruchnik, Janusz D. Fidelus, Ewelina Gacka, Krzysztof Kwoka, Julia Pruchnik, Adrianna Piejko, Łukasz Usydus, Leszek Zaraska, Grzegorz D. Sulka, Tomasz Piasecki, Teodor P. Gotszalk

**Affiliations:** 1 Department of Nanometrology, Wrocław University of Science and Technology, Janiszewskiego 11/17, 50-370 Wroclaw, Poland; bartosz.pruchnik@pwr.edu.pl (B.C.P.); ewelina.gacka@pwr.edu.pl (E.G.); krzysztof.kwoka@pwr.edu.pl (K.K.); julia.pruchnik@pwr.edu.pl (J.P.); adrianna.piejko@pwr.edu.pl (A.P.); tomasz.piasecki@pwr.edu.pl (T.P.); teodor.gotszalk@pwr.edu.pl (T.P.G.); 2 Time and Length Department, Central Office of Measures, Elektoralna 2, 00-139 Warsaw, Poland; 3 Electricity and Radiation Department, Central Office of Measures, Elektoralna 2, 00-139 Warsaw, Poland; lukasz.usydus@gum.gov.pl; 4 Department of Physical Chemistry and Electrochemistry, Faculty of Chemistry, Jagiellonian University, Gronostajowa 2, 30-387 Krakow, Poland; zaraska@chemia.uj.edu.pl (L.Z.); sulka@chemia.uj.edu.pl (G.D.S.)

**Keywords:** electrical measurements, four-point measurement, nanowires, nanowire resistance measurement, nanomanipulation, AFM, ZnO

## Abstract

The measurement method, which utilizes nanomanipulation of the nanowires onto a specially prepared substrate, was presented. It introduced a four-point resistance measurement setup on a chip suited for scanning probe microscopy measurements, integrating connectors and a nanowire specimen. A study on the resistance and resistivity of the thermally post-treated ZnO nanowires at 200 °C and 300 °C in air showed the dependence of these electrical parameters on the annealing temperature. The investigations of the electrical properties of blocks built on the basis of nanowires and their related devices could provide a useful guide not only for designing, fabricating and optimizing electromechanical nanodevices based on nanowires but also for their safe operation in future electronic applications.

## 1. Introduction

In recent years, nanowires (NWs) have found applications in contemporary electronics, for example in MOSFET transistors, energy harvesting devices and photovoltaic circuits. Knowledge of the electrical parameters of NWs is essential for the use of these structures in electronic circuits [1], which is a promising area, as NWs exhibit various interesting mechanical and mechanoelectrical properties [2,3]. The development of repeatable and adequate electrical measurement methods is still in progress, as both nanomanipulation and nanometrology are relatively new areas of research.

NWs are an emerging branch of nanotechnology; therefore, both their measurements and applications are still in broad development. The resistance of NWs is one of their crucial properties, determining suitability for sensing applications; depending on the application, it may vary due to various environmental influences. Hence, resistance measurements are among the most sought-after. Measurement versatility is therefore an important metrological task.

One of the basic electrical quantities describing this type of element and its resistance may be current–voltage characteristics (I–V). Due to their size, measurements made on NWs (especially on a single one) are not a simple task, require a very precise measuring system and can be time-consuming. Furthermore, for purposes of correlative measurements, the chosen specimen has to withstand the measurement.

The most straightforward approach to measuring the electrical properties of a single NW employs two probes that are in ohmic contact with the NW, i.e., no energy barrier is created [4,5]. A scanning electron microscope (SEM) can be used for probe positioning [6]. The sample is placed on an insulating substrate in order to avoid current leakage. A small current is then applied to the NW, which flows from one probe tip to the other, and the drop of voltage across the specimen is measured, which yields the I–V characteristics. With those, one can calculate the resistance and resistivity of the sample. The downside of a two-point setup is that the resistances of contacts and/or connections have an influence on the measurement result [4,7]. It is especially visible when measuring NWs made of highly conductive materials, where it can lead to significant voltage measurement errors. This disadvantage can be avoided by using a so-called four-point measurement setup. In such a case, there are two pairs of probes: one for current injection and another for voltage measurement. If the intrinsic resistance of a voltage measuring device is large, one can assume that the current flow across its circuit is negligible, and therefore there is no voltage drop on probe-to-NW contact, which leads to more precise voltage measurements across the NW. Technique is especially important in material engineering for measurements of novel materials with yet unknown electrical properties [8]. Despite its adequacy in the field, this method is not widely applied to measurements of NWs, primarily due to the issues described further.

Probes that are used to make contact with a NW can be in the form of sharp tips, but that is only one solution. Other approaches include sets of probes manufactured as microchips on which the test sample is placed. These are either deposited on an isolating substrate or sometimes suspended in air or vacuum, which makes for quite a complex nanosystem but ensures better thermal and electrical isolation [4]. Apart from current injection, some nanoprobes can be used for heating NWs and then observing how their electrical parameters change with temperature. Some setups, on the other hand, allow for mechanical manipulation (stretching, bending) of the test sample, which enables, for example, the analysis of piezoelectric phenomena in semiconducting NWs [9].

An alternative approach to those described above is measurement with scanning probe microscopy (SPM) techniques [5]. One of the most common SPMs is the atomic force microscope (AFM). This method relies on a probe in the form of a tip that scans NWs with nanometric resolution [4]. The difference from the two- or four-point measurement is that this approach can be used to measure the electrical properties of NWs embedded inside structures such as matrices. To obtain I–V curves, one can inject electrical current into a NW and use the conductive tip of an AFM to measure the voltage difference at different positions across the test sample. From several I–V curves and knowing their geometrical dimensions, it is possible to obtain NWs’ electrical resistivity.

Measurements of the electrical properties of NWs can be performed in a similar way using scanning tunneling microscopy (STM). Unlike AFM, this method does not require physical contact between the probe tip and the test sample. It is based on the concept of quantum tunneling, which occurs when there is a voltage difference between the tip of a conductive probe and the surface under examination. After bringing the tip close enough to the surface, electrons start to tunnel through the vacuum between them, which results in a current flow. With a bias voltage applied to the ends of a NW (e.g., by putting it between two electrodes), one can measure voltage differences along its length by scanning with the probe tip. Thus, the I–V characteristics of the sample can be obtained, and then, using the geometrical dimensions, resistivity and resistance can be calculated. STM measurements are more time-consuming and can be used mostly to characterize structures made of conducting materials [4,10].

Kelvin probe force microscopy (KPFM) is another type of SPM technique that can be used to measure the electrical properties of NWs [6]. Using this method, one can probe the contact potential difference (the difference between the work functions of the tested sample and the conductive tip). The voltage drop along the length of NW is estimated by subtracting KPFM signals obtained while the specimen is biased or unbiased [11]. To yield the I–V curves of the specimen, the value of the current flowing through the NW and the length along which the voltage drop have to be measured. Vinaji et al. [11] presented example results of voltage and resistance measurements using KPFM on GaAs NWs, where a resistance of about 57 kΩ was measured for over 3.2 µm of a single NW with a diameter of circa 240 nm.

The electrical properties of single semiconducting NWs can also be characterized with scanning photocurrent microscopy (SPCM). In this case, the test sample is placed on a microchip with bias electrodes on an insulating substrate. The laser beam from the SPCM probe generates an excess of carriers and locally induces an electrical current (photocurrent) [4]. Plotting these current values against the bias voltage yields the I–V characteristics of NWs. It can be noticed that this technique is somewhat similar to SPM, with differences in the scanning mechanism and current excitation. Moreover, it cannot be used for the characterization of NWs made of certain materials. InAs NWs have been measured by Chu et al., using SPCM [12]. A resistance value of 22 kΩ was obtained for 5.8 µm long NWs with about 200 nm in the diameter.

The downside of all probing methods is the necessity of exact localization and then sustaining stable electrical contact throughout the measurement [13]. Instead of probing the NW sample, it can be deposited on a specially prepared substrate, incorporating conductive paths that serve as electrical contacts. Substrates fabricated with CMOS techniques were already used, originally with the NW put on top of all four contacts [14]. They, however, have limitations: contacts fabricated with the use of photolithographic methods are limited to ~1 μm of characteristic dimensions, rendering the whole four-point structure at least 7 μm long and often much bigger. A nanowire, to be measured properly, has to achieve that length, which may be impossible for some materials.

This challenge may be overcome with the application of higher-resolution lithography techniques (extreme UV lithography—EUL, electron beam lithography—EBL) or localized methods. Especially interesting is focused electron beam induced deposition (FEBID), since it allows for the deposition of material regardless of the substrate material. It can be applied to the substrate with NWs already delivered. Fabrication of contacts to the single NW localized on the surface was already performed by [15] or [16] on a broad substrate. However, in a four-point measurement setup, resistances of contacts do not influence the measured value; they can influence the resolution of measurement, especially long FEBID paths, which may exceed the resistance of NW by orders of magnitude.

To limit the length of contacts, NWs may be brought to the desired area. The first historically used method was fabrication on surface artifacts, which created predictable NWs [17]. A more ubiquitous method is, however, nanomanipulation of NW delivered from other substrates [13,18]. In that case, limitation is only given by the mechanical strength of a single NW.

Various already introduced methods covered the usage of probes of dimensions adequate to the NWs, special substrates with conductive parts or the deposition of electrical setup around the localized specimen. To elude limitations imposed by each method we introduce a combinatory approach, in which the NW is connected to prefabricated contacts with material grown by focused electron beam induced deposition (FEBID). The precise nature of the contact formed between ZnO and a FEBID material is not yet described; however, it may be omitted in the four-point setup. Simultaneously, deposited contacts have a length shorter than the NW itself. Furthermore, the NW is delivered to the desired localization with nanomanipulators; therefore, its orientation and connection are freely adaptable. A sample created in this way is fully transferable between measurement systems. Using the method, we present measurements of the resistivity of ZnO nanowires and its change due to annealing, leading to a changing degree of crystallization.

## 2. Materials and Methods

The measurement method was based on the fabrication of persistent connections between the measured nanostructure and pre-manufactured electrical contacts. NWs were brought to the measurement locations with needle nanomanipulators. Contacting was performed using FEBID material. The strength of the contact was then tested using a nanomanipulator. Such a prepared sample was measured first in a 2-point and then in a four-point electrical measurement setup. Each of the experimental setup parts is elaborated on in further subsections. The novelty of the method lies in the direct placement of the NW combined with the fabrication of integrated electrical and mechanical connections. Except for measurements of basic parameters (such as resistivity), the setup allows for much more sophisticated applications for the search of transducing properties thanks to the direct localization and repeatability of the nanosetup.

### 2.1. Nanowires

**Figure 1:**
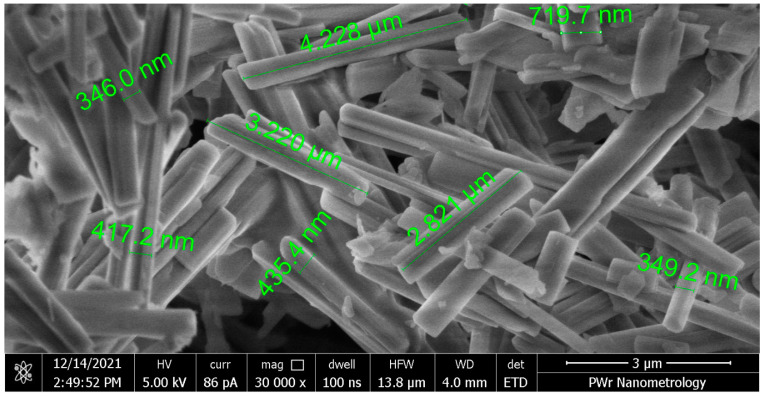
Typical NWs detached from original substrate mechanically. To deliver NWs to the surface, they were mechanically detached from the original substrate and delivered to the measurement point with the use of a stainless steel stylus, without the use of solvent or transfer medium. The length of nanowires after thermal treatment is, in this case, ca. 20 µm. However, as can be seen in Figure 1, much shorter wires were used for the measurements since they broke during their detachment from the metallic substrate. As NWs were placed singularly with nanomanipulators, there was no need for further atomization of the granules. Out of all annealed samples, two subtypes of ZnO NWs were chosen for measurements: annealed in air at 200 °C and 300 °C. With annealing, the degree of ZnO NW crystallization increased, so we obtained ZnO NWs with different degrees of crystallinity. The changes in crystallinity caused by the thermal treatment of the nanowires obtained during anodization were studied in our previous works [20,21]. According to these results, crystallinity change (defined as shift in sizes of crystals) should occur between two selected samples. ZnO NW annealed at 300 °C in air should have undergone a crystalline phase change to Wurzite structure, which would affect the conductivity of the NW [22].

In the experiments, zinc oxide (ZnO) NWs were examined. ZnO NWs are promising materials due to their predicted properties in fields such as piezoelectricity or photovoltaics. Therefore, the development of a versatile and transferable measurement setup for NW with the possibility of combining resistance measurements with PV effects or piezoelectric effects is key to the proper description of the phenomena. They were manufactured in dense arrays with typical diameters in the range of 100–200 nm and lengths of several or a dozen µm. They were obtained by using one-step anodic oxidation of metallic Zn foil in a 10 mM sodium bicarbonate electrolyte and thermal post-treatment. All anodization processes were carried out at room temperature in a homemade Teflon cell with the Zn substrate (99.95% Goodfellow, thickness 0.5 mm) placed directly on a conductive support and a Pt grid working as a cathode. Further, samples were rinsed with deionized water multiple times, dried and then annealed in air at different temperatures in the range from 100 to 300 °C for 2 h, using a muffle furnace with a heating rate of 2 °C min^−1^. Details of the method of obtaining ZnO NW can be found in [19,20]. The characteristic morphology of an array of NWs is presented in Figure 1.

### 2.2. Measurement Substrates

**Figure 2:**
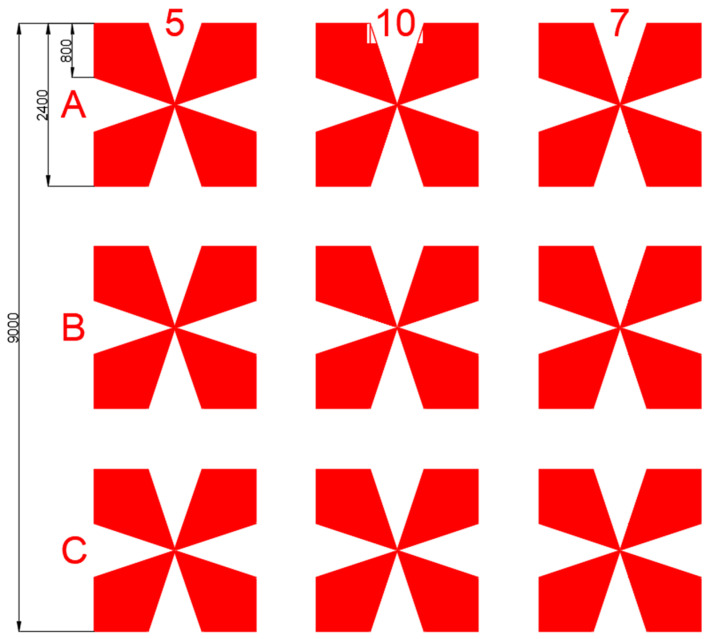
Photolithographic mask of metallic contacts (red) on a single chip—9 devices arranged by 3 of each gap distance. The mask is marked with numbers denoting gap lengths in each column and letters describing rows for clear allocation.

Prepared substrates consisted of thin-film metal paths (platinum or gold) over silicon oxide (SiO_2_). Paths were organized in groups of four, narrowing toward the contact area, in which they were separated by gaps of 5, 7 or 10 μm. Nine such contacting structures were gathered on one chip. More details are in Figure 2.

**Figure 3:**
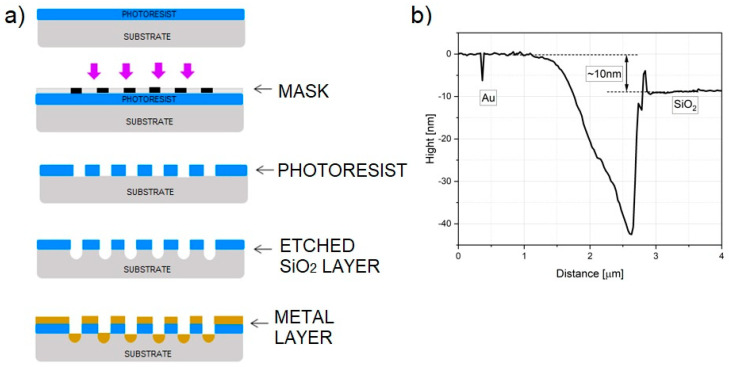
(**a**) Scheme of lift-off process of fabrication of buried planar contacts with successive steps; (**b**) substrate profile with marked height of contacts above the SiO_2_ layer.

The manufacturing of structures was performed using CMOS techniques. The SiO_2_ layer was developed on the silicon surface with annealing in an oxygen atmosphere. A metal layer was further deposited by molecular beam epitaxy (MBE). Patterning was made with photolithography using the lift-off process (Figure 3a). A negative resist was used for the lithography process, which was developed on a substrate and then exposed to UV light. The substrate with the developed pattern was etched in a mixture composed mainly of hydrofluoric acid (BHF) to etch the SiO_2_ layer under the contact area. In this process, the non-UV-exposed areas of the SiO_2_ layer were etched to a depth of about 100 ± 10 nm. A metal layer (~120 nm) was then deposited. To make the exposed pattern of contacts more visible, the residual resist on the substrates was washed with solvent (acetone). The end result is the substrate with partially buried planar contacts to a height of about 10 nm above the SiO_2_ surface.

Contacts ended with 0.8 mm pads for contacting electrically. Although the metal layer thickness (approximately 10 nm) prevented wire bonding (Figure 3b), it was still possible with a conductive adhesive (silver DAG) or needle probes. Substrates were subsequently mounted on PCB hubs for soldering the wires and ease of handling.

### 2.3. Nanowires Contacting

**Figure 4:**
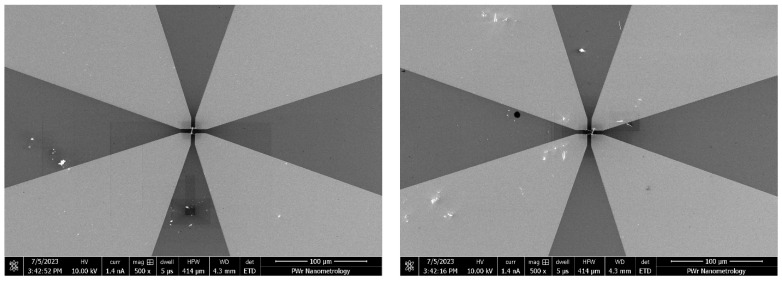
NWs deposited between electrical contacts (brighter) over SiO_2_ (darker). NW debris can be observed in close proximity.

NWs were imaged with SEM (FEI Helios Nanolab 600i) in order to localize the promising specimen. We searched for a singular continuous NWs with a length of above 5 μm. Found NWs were brought to the location between contacts with the EasyLift nanomanipulator. Regular procedure is to bond the NW with the manipulator’s tip with FEBID material, which is then cut off using focused ion beam (FIB). The inherent property of FIB is, unfortunately, heavy doping of incident regions, which would then influence the conductivity of the measured material [23]. Therefore, NWs were attached to the nanomanipulator needle electrostatically. Despite being more whimsical, this approach provides adhesion force good enough to deliver the NW few hundreds of micrometers to the desired location (Figure 4). The gaps on the substrate were manufactured in order to serve various sizes of nanostructures. Since the gap size was not perceived to have any influence on measured value, gaps were chosen appropriately for the selected NW length.

**Figure 5:**
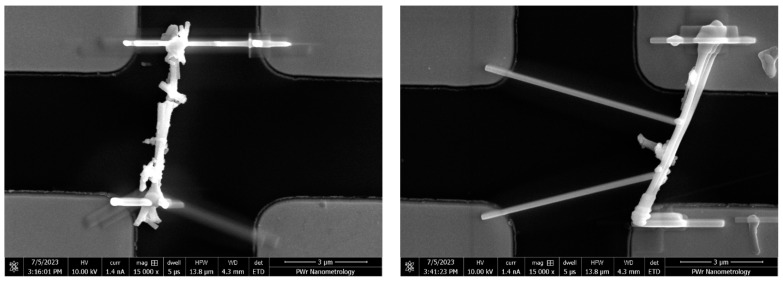
ZnO NWs annealed at 200 °C (**left**) and 300 °C (**right**) secured on the surface of the measurement substrate.

There, the NWs were bound to the surface with FEBID material—specifically platinum–carbon (PtC) nanocomposite was used. The theoretical conductivity of the material is at the level of a few ohm centimeters, but the contact resistance between PtC and ZnO is unknown. To disregard this issue and measure the resistance of only the ZnO material, NWs were connected in a four-point setup (Figure 5). FEBID paths were deposited to be several tens of nm high. They differed in length, which does not make a difference in DC measurements of resistance.

### 2.4. Electrical Measurements

Electrical properties were measured in a predesigned setup, which consisted of a Keithley2603 source-measuring unit (SMU) connected with triaxial cables to four probes connected directly to the substrate. For additional noise-proofing, both samples and probes were enclosed within a Faraday cage. To avoid excessive currents or electrostatic discharge effects, an additional resistance of 10 MΩ was included in the current path.

**Figure 6:**
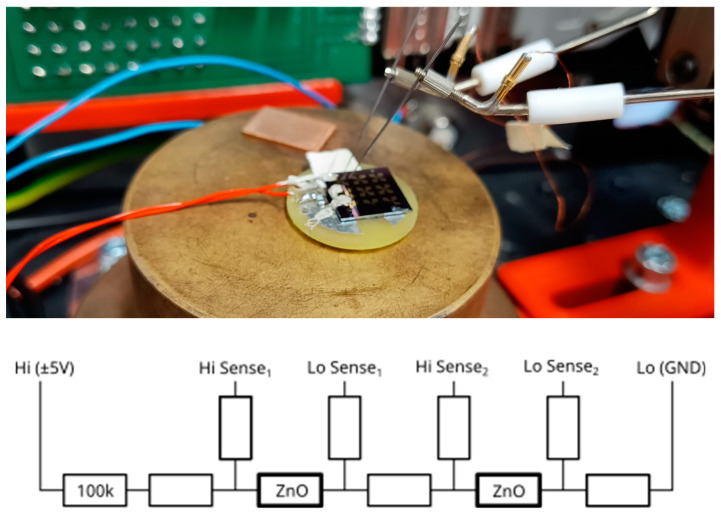
Four-point measurement setup and schematic “sense” contacts, which may be rearranged easily; therefore, they are depicted in multiples.

Among the used probes, two were needle probes on micromanipulators (hence easily rearrangeable), and the other two were permanently connected through a PCB sample board. Probes were soldered to the base PCB sample board, while direct connections to the chip surface were made with silver DAG. To make the setup stable, two permanent contacts were used as current probes and joined in series with both NWs to form a current path (Figure 6). Movable probes were used as sense (voltage) probes.

The measurement was conducted and controlled by software Impedancer2, developed within the Department of Nanometrology. It allowed for planning the measurement scenarios, including current and voltage sweeps with set limit values. It was used for the automated measurement of I–V curves.

## 3. Results

The purpose of the measurement was to assess the resistivity of the material of which the nanowires consisted. The next step was the measurement of the geometrical dimensions of the conductor and the resistance.

**Figure 7:**
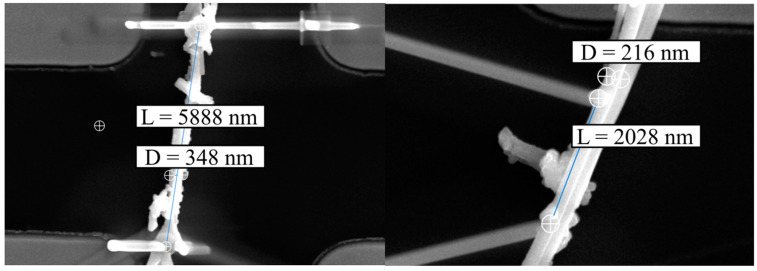
ZnO NW dimensions (D—diameter, L—length) measured from SEM images.

The determination of geometrical dimensions required the definition of the measured structure. The NWs bound to the surface consisted of a few wires held together adhesively. The pre-annealed NW was a single, continuous NW surrounded by ZnO debris; annealed one was two or three joined continuous wires. For that purpose, FEBID was carefully deposited only to connect one of them. Finally, geometry was determined by imaging with SEM (Figure 7), assumed to be a circular cross-section of the NW, and the length was measured between axes of closer (voltage) contacts.

In order to remove any potential capacitive effects, the measurement of resistance was performed as a DC voltage sweep between 0 and 5 V, with voltage increasing and dropping. Furthermore, measurements were repeated a few times to eliminate stochastic effects. Eventually, both ZnO NWs were measured in the same manner.

**Figure 8:**
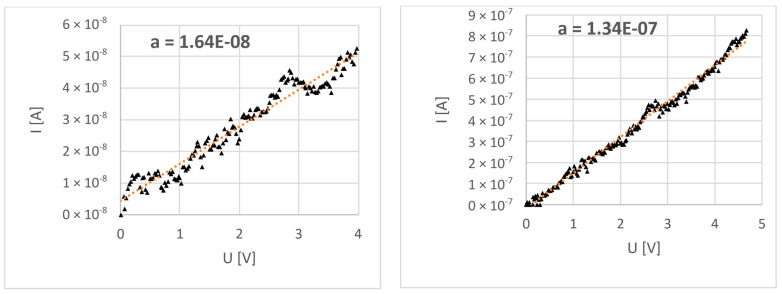
ZnO NW I–V characteristics from the measurement in a 4-point setup. Date points depicted with triangle points, dotted line—linear regression.

Measurements resulted in I/V curves that were linear at the measured range (Figure 8). Linear regression was applied to determine the slope of the characteristic, which is directly related to the conductance of the measured element. Therefore, 200 °C NW reached a resistance (*R*) value of 74.8 MΩ, while that of the 300 °C NW was 6 MΩ. For these values and taking into account the measured dimensions of the wires (length *l* and diameter *D*), resistivity was calculated as follows:

$$\rho =R\frac{\pi D^{2}}{4l}$$

and the following resistivity values were determined: 200 °C NW, 3.9 Ωm; 300 °C NW, 0.55 Ωm. All experimental values, along with measurement errors are presented in Table 1. Error values were based on measurement resolution and the standard deviation of the series. The complex error was calculated based on the exact differential method [24].

## 4. Discussion

The measured resistivity is within the limits of previously reported values [25]. Exact parameters may vary depending on the technological process, annealing environment, post-treatment, material composition, doping and many other parameters [26]. In the work of Marlene N. Cardoza-Contreras et al. [16], the authors report the dependence of the resistance of ZnO NW on its diameter. Electrical resistances of single ZnO nanowire obtained from I–V curves were 2.05 × 10^5^ Ω, 3.58 × 10^4^ Ω and 44.38 × 10^3^ Ω for diameters of 800 nm, 700 nm and 500 nm, respectively. The resistance value for the ZnO NW with a diameter of 500 nm corresponds well with the NW tested by us annealed at 200 °C (~75 MΩ) with diameter of approx. 350 nm. On the other hand, the obtained resistance values of ZnO nanowires are slightly higher than those reported by Wang et al. [27], who, for the ZnO NW with a length of 0.2 mm and a diameter of 40 nm, obtained a resistance in the range from 16 kΩ to 16 MΩ. He et al. [28] state in their work that the obtained resistivity of ZnO nanowires from 10^−2^ to 10 Ωcm (10^−4^ to 10^−1^ Ωm) depends on the contacts on the electrodes and the concentration of oxygen vacancies. In our case, the annealing of the samples at different temperatures most likely resulted in differences not only in the increased degree of crystallinity but also in the reduced number of defects [28].

The main contribution of the work is the measurement method, which utilizes nanomanipulation of the nanowires onto a specially prepared substrate. The achieved results confirm the predicted parameter shift due to the increasing degree of crystallization during annealing. What is more, the achieved measurement error is calculable and remains within the limit of 20% of the measured value. It can be improved even further with more careful measurement of dimensions, as the limitation of the used SEM is about 5 nm; other methods may be applied as well, e.g., AFM. Resistance resolution may be improved with longer integration times and a reduction in external noise.

Measurement nanosetup, being applied in a repeatable manner to the NWs, enables measurement of basic properties, one of which is resistivity. Thanks to the direct localization of the measured element, combined with the fabrication of integrating electrical and mechanical connections, much more sophisticated applications might be successfully applied, i.e., the search for transducing properties.

The prepared sample is flat in the sense that no connection exceeds the height of the NW in its proximity. That feature makes it suitable for various SPM methods to be applied. NW is placed on a substrate but fixed to it only at a few points, which makes it mechanically examinable. Correlative microscopy methods can be applied to determine not only single-phenomenon (mechanical, electrical, electronic and thermal) properties but also to search for transducing properties of the NW.

## 5. Conclusions

In summary, we have shown the measurement method, which utilizes nanomanipulation of the nanowires onto a specially prepared substrate. The investigations of the electrical properties of ZnO building blocks (on the basis of nanowires) and their related devices will provide a useful guide not only for designing, fabricating and optimizing electromechanical nanodevices based on ZnO nanomaterials but also for their safe operation in future electronic applications.

The applied method proved to be accurate in accordance with the theoretical predictions concerning the resistivity of the ZnO nanowires with different rates of crystallization. In the given setup, it is possible to correlate electrical measurements with measurements of other quantities. Primarily, with a four-point fixing mechanism, it becomes viable to examine electromechanical characteristics.

Possibility to introduce the NW with an electrical measurement setup to SPM in the arising possibility, which would lead to true correlative, single specimen measurements of nanostructures.

## Figures and Tables

**Table 1:** Results of measurements with error calculations.

Annealed Sample	Length (nm)	Diameter (nm)	Resistance (MΩ)	Resistivity (Ωm)
200 °C	5888 ± 50	348 ± 20	74.8 ± 1.25	3.9 ± 0.46
300 °C	2028 ± 50	216 ± 20	6 ± 0.11	0.55 ± 0.11

## Data Availability

Measurement data are available upon request.

## References

[1] Bieniek T., Janczyk G., Janus P., Grabiec P., Nieprzecki M., Wielgoszewski G., Moczała M., Gotszalk T., Buitrago E., Badia M.F.-B. (2013). Silicon nanowires reliability and robustness investigation using AFM-based techniques. Electron. Technol. Conf..

[2] Jang S., Sung J., Chang B., Kim T., Ko H., Koo K.-I., Cho D.-I. (2018). Characterization of the Piezoresistive Effects of Silicon Nanowires. Sensors.

[3] Hur S., Lee K.-H., Hahn Y.-B., Kim W.-D., Choi H. Power generation using piezoelectric ZnO nanowires for nano-scale devices. Proceedings of the 10th IEEE International Conference on Nanotechnology.

[4] Rojo M.M., Calero O.C., Lopeandia A.F., Rodriguez-Viejo J., Martín-Gonzalez M. (2013). Review on measurement techniques of transport properties of nanowires. Nanoscale.

[5] Michels T., Rangelow I.W. (2014). Review of scanning probe micromachining and its applications within nanoscience. Microelectron. Eng..

[6] Ru C., Zhang Y., Sun Y., Zhong Y., Sun X., Hoyle D., Cotton I. (2010). Automated Four-Point Probe Measurement of Nanowires Inside a Scanning Electron Microscope. IEEE Trans. Nanotechnol..

[7] Janesch J. (2013). Two-Wire vs. Four-Wire Resistance Measurements: Which Configuration Makes Sense for Your Application?.

[8] Nägelein A., Liborius L., Steidl M., Blumberg C., Kleinschmidt P., Poloczek A., Hannappel T. (2017). Comparative analysis on resistance profiling along tapered semiconductor nanowires: Multi-tip technique versus transmission line method. J. Physics Condens. Matter.

[9] He H., Hsin L., Liu J., Chen J., Wang L. (2009). Piezoelectric Gated Diode of a Single ZnO Nanowire. Adv. Mater..

[10] Ramanayaka A.N., Kim H.-S., Tang K., Wang X., Silver R.M., Stewart M.D., Pomeroy J.M. (2018). STM patterned nanowire measurements using photolitographically defined implants in Si(100). Sci. Rep..

[11] Vinaji S., Lochthofen A., Mertin W., Regolin I., Gutsche C., Blekker K., Prost W., Tegude F.J., Bacher G., Caldas M. (2010). Local Electrical Analysis of a Single Semiconductor Nanowire by Kelvin Probe Force Microscopy. AIP Conf. Proc..

[12] Cheng-Hao C., Ming-Hua M., Che-Wei Y., Hao-Hsiung L. (2019). A New Analytic Formula for Minority Carrier Decay Length ex-traction from scanning Photocurrent Profiles in Ohmic-Contact Nanowire Devices. Sci. Rep..

[13] Lin R., Bammerlin M., Hansen O., Schlittler R.R., Bøggild P. (2004). Micro-four-point-probe characterization of nanowires fabricated using the nanostencil technique. Nanotechnology.

[14] Cronin S., Lin Y., Koga T., Sun X., Ying J., Dresselhaus M. Thermoelectric investigation of bismuth nanowires. Proceedings of the International Conference on Thermoelectrics (ICT).

[15] Miccoli I., Edler F., Pfnur H., Tegenkamp C., Prete P., Lovergine N. (2018). Surface-mediated electrical transport in single GaAs nanowires, 2015 1st Work. Nanotechnol. Instrum. Meas. NANOFIM.

[16] Cardoza-Contreras M.N., Romo-Herrera J.M., Ríos L.A., García-Gutiérrez R., Zepeda T.A., Contreras O.E. (2015). Single ZnO Nanowire-Based Gas Sensors to Detect Low Concentrations of Hydrogen. Sensors.

[17] Menke E.J., Brown M.A., Li Q., Hemminger J.C., Penner R.M. (2006). Bismuth Telluride (Bi_2_Te_3_) Nanowires: Synthesis by Cyclic Electrodeposition/Stripping, Thinning by Electrooxidation, and Electrical Power Generation. Langmuir.

[18] Mølhave K., Wich T., Kortschack A., Bøggild P. (2006). Pick-and-place nanomanipulation using microfabricated grippers. Nanotechnology.

[19] Zaraska L., Mika K., Syrek K., Sulka G.D. (2017). Formation of ZnO nanowires during anodic oxidation of zinc in bicarbonate electrolytes. J. Electroanal. Chem..

[20] Zaraska L., Mika K., Hnida K.E., Gajewska M., Łojewski T., Jaskuła M., Sulka G.D. (2017). High aspect-ratio semiconducting ZnO nanowires formed by anodic oxidation of Zn foil and thermal treatment. Mater. Sci. Eng. B.

[21] Mika K., Wiercigroch E., Pisarek M., Kozieł M., Majda D., Lytvynenko A.S., Sulka G.D., Zaraska L. (2023). Nanostructured films formed on Zn during anodic oxidation in different carbonate-based electrolytes. Appl. Surf. Sci..

[22] Paiman S., Ling T.H., Husham M., Sagadevan S. (2020). Significant effect on annealing temperature and enhancement on structural, optical and electrical properties of zinc oxide nanowires. Results Phys..

[23] Cronin S.B., Lin Y.M., Koga T., Ying J.Y., Dresselhaus M.S. (1999). Transport Measurements of Individual Bi Nanowires. MRS Proc..

[24] Farrance I., Frenkel R. (2012). Uncertainty of Measurement: A Review of the Rules for Calculating Uncertainty Components through Functional Relationships. Clin. Biochem. Rev..

[25] Lord A.M., Maffeis T.G., Walton A.S., Kepaptsoglou D.M., Ramasse Q.M., Ward M.B., Köble J., Wilks S.P. (2013). Factors that determine and limit the resistivity of high-quality individual ZnO nanowires. Nanotechnology.

[26] Schlenker E., Bakin A., Weimann T., Hinze P., Weber D.H., Gölzhäuser A., Wehmann H.-H., Waag A. (2008). On the difficulties in characterizing ZnO nanowires. Nanotechnology.

[27] Yi G.-C., Wang C., Park W.I. (2005). ZnO nanorods: Synthesis, characterization and applications. Semicond. Sci. Technol..

[28] He J.H., Lao C.S., Chen L.J., Davidovic D., Wang Z.L. (2005). Large-Scale Ni-Doped ZnO Nanowire Arrays and Electrical and Optical Properties. J. Am. Chem. Soc..

